# Neurodevelopmental Outcome of Infants with Gastroschisis at One-Year Follow-Up

**Published:** 2015-04-01

**Authors:** Vishal Gupta, Amit Trivedi, Karen Walker, Andrew J A Holland

**Affiliations:** 1 Grace Centre for Newborn Care, The Children’s Hospital at Westmead, Westmead NSW 2145, Australia; 2 2Douglas Cohen Department of Paediatric Surgery, The Children’s Hospital at Westmead, Sydney Medical School, The University of Sydney, New South Wales, Australia

**Keywords:** Gastroschisis, Newborn, Neurodevelopment

## Abstract

Objective: Gastroschisis is a congenital malformation of the abdominal wall and may be associated with significant neonatal mortality and morbidity. The primary objective of this study was to describe the neurodevelopmental outcomes of neonates with this condition.

Methods: Medical records of all neonates admitted with a diagnosis of gastroschisis to a tertiary surgical unit from October 2006 to August 2011 were retrospectively reviewed. Demographic and clinical variables were collated along with developmental assessment results at one-year follow-up. Developmental assessment results were compared with case matched healthy control neonates of similar gestational age and birth weight.

Results: Of 20 patients in the study, 16 had simple and four had complex gastroschisis. Mean birth weight was 2.29 kg with a mean gestational age of 35.7 weeks. The majority of neonates underwent primary surgical repair, while 15% had a silo followed by surgical repair. Neonates with gastroschisis did not significantly differ from the control group in neurodevelopmental outcomes. Receptive and expressive language delay was found in gastroschisis is attributable to small for gestational age rather than the malformation per se.

Conclusions: These data suggest that neurodevelopmental outcomes at one year of age in children with gastroschisis were associated with being small for gestational age rather than the malformation.

## INTRODUCTION

Gastroschisis (GS) is a full thickness, para-umbilical abdominal wall defect associated with evisceration of the intestine.[1] The incidence of GS has been reported as 1-5/ 10,000 live births and is increasing worldwide.[1-4] Nulliparous women and women under 20 years of age have a higher incidence of affected neonates.[5-9] Approximately 50% of neonates with GS are born prematurely and 67% are small for gestational age (SGA).[6] Prematurity, intrauterine growth restriction and nutritional issues in the immediate postnatal period potentially impact on growth and neurodevelopment.[10]

The majority of studies examining the outcomes in GS have focused on in-hospital end-points such as commencement of feeds, days on ventilation, duration of Total Parenteral Nutrition (TPN), TPN associated cholestasis and length of stay in the hospital.[9,11,12] There remains a paucity of data reporting the neurodevelopment outcome of these neonates: the few that have been published have conflicting results, with both impaired [11] and normal development.[12,13] The aim of this study was to determine the neurodevelopment outcome of neonates with GS at one year of age.

## MATERIALS AND METHODS

Medical records of all neonates admitted with a diagnosis of gastroschisis to a tertiary surgical unit from October 2006 to August 2011 were retrospectively reviewed. All the neonates were transferred after delivery to the tertiary unit for surgical repair. Data were collected from the hospital’s electronic records and discharge summaries. Bayley Scales of Infant and Toddler Development (BSID-III)® [14] assessment forms were used to evaluate them at follow up.

Patient data included maternal age at birth, gestational age at delivery, mode of delivery, APGAR score at one and five minutes and birth weight. Type of GS and surgical repair were recorded as independent variables. The outcome variables were duration of mechanical ventilation in days, age in days at commencement of feeds, number of days to full enteral feeds, duration of TPN, neuroimaging, TPN cholestasis and gastro-intestinal complications. 

All patients with GS have been enrolled in a developmental follow-up clinic since October 2006 with assessments performed using the BSID-III. Domains assessed by BSID-III included Cognition, Communication (receptive and expressive), Gross motor and Fine motor subtests.

The scaled scores were obtained and classified as below average, average and above average.[15] Case-control analysis was performed to evaluate development at one year of age. Each patient with GS was matched to a control patient of the same gestational age (in weeks) and birth weight at one year of age. Control group comprised of healthy neonates who also formed the control group of another published study. [16] The study was approved by the local health network ethics committee.

**Definitions and statistical analysis:**

1. Preterm: less than 37 weeks of gestation age at delivery.

2. Small for gestation (SGA): Birth weight less than 10th centile for gestational age.

3. Simple GS: Gastroschisis with uncomplicated repair.

4. Complex GS: Gastroschisis complicated by atresia, stenosis and bowel resection.

5. Total parenteral nutrition (TPN) cholestasis: Greater than 30 µmol/L of conjugated bilirubin in neonates who have received total parenteral nutrition.

6. Gravida status: Primigravida being women pregnant for the first time and multigravida being women who has conceived one or more times in the past.

Data were analysed using IBM SPSS statistics (version 17; SPSS, Chicago, IL). ANOVA and t-test was used to compare developmental scores.

## RESULTS

Twenty neonates with GS were enrolled in the study between October 2006 and August 2011. The mean gestational age was 35.7 weeks, and the mean birth weight was 2.29kg (Table 1). An antenatal diagnosis by foetal ultrasound was available in all neonates.

**Figure:**
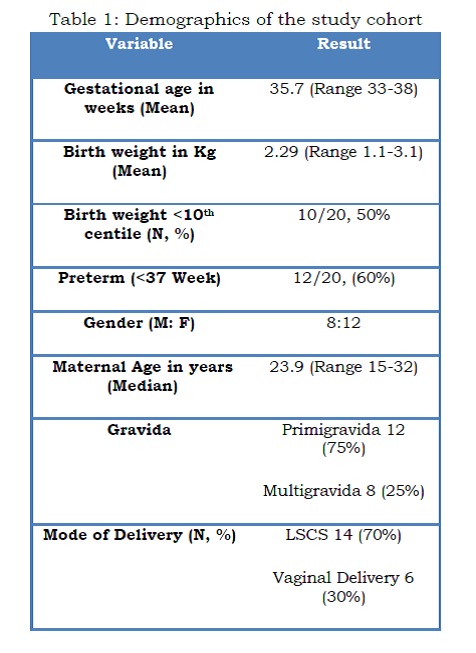
Table 1: Demographics of the study cohort

The majority, 16 out of 20 (80%), had simple GS, while four neonates (20%) had complex GS None of the neonates had any associated congenital anomalies. Primary closure was achieved for the majority of neonates, 14/20 (70%) shortly after admission. Three infants required a Silo reduction and three neonates required multiple stage operations in view of associated intestinal atresia and postoperative strictures. Three neonates had TPN cholestasis although none led to chronic liver disease. The median duration of ventilation was 3.5 days (0-43) while the median duration on TPN was 21.5 days (8 -124). The median time to start feeds was 7.5 days (2 to 98 days) and the median stay in the hospital was 27.5 days (12-218) (Table 2).

**Figure:**
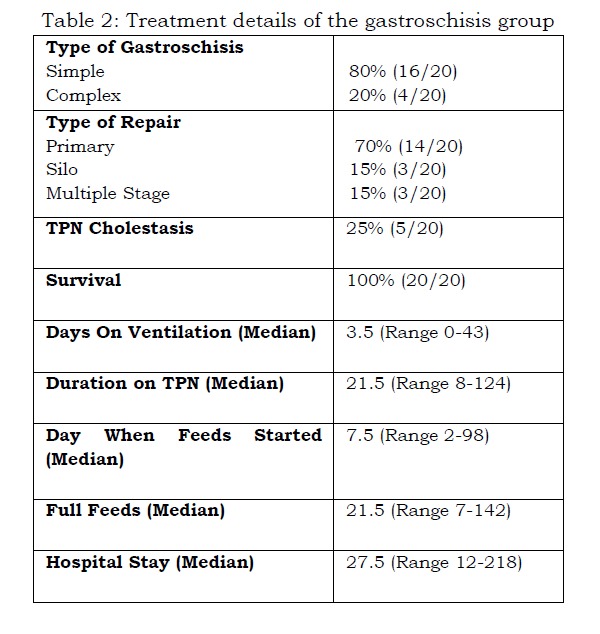
Table 2: Treatment details of the gastroschisis group

Simple GS was managed by primary repair or Silo (16/16) while the majority (3/4) of complex GS required multiple-staged repair. Infants with complex GS had significantly longer duration of ventilation, time to feeds commenced, longer length of stay and time to full feeds (Table 3). 

**Figure:**
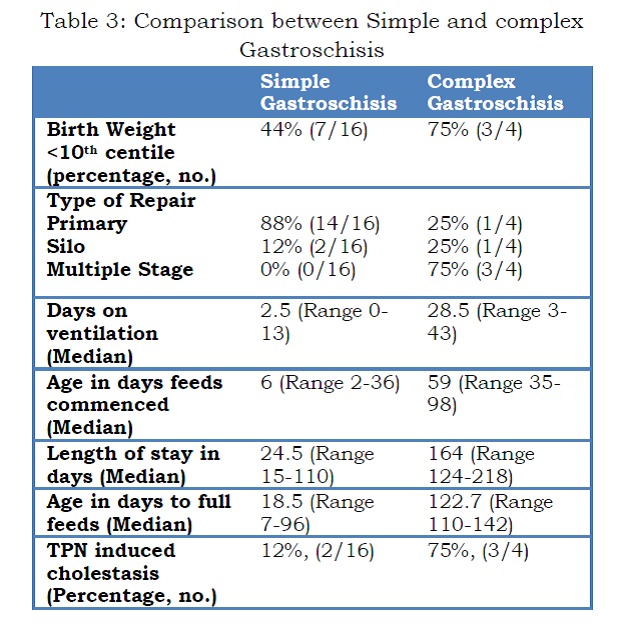
Table 3: Comparison between Simple and complex Gastroschisis

Growth and development were assessed at one year of age. 18/20 infants had follow-up data at a corrected mean age of 13 months (11-16 months). 28% of neonates had a weight below 10th centile (SGA) at one year of age compared with 50% at birth. Of the four neonates with complex GS, three were less than 10th percentile for weight at birth (SGA) and these infants remained growth restricted at their one-year follow up.

There were no statistically significant differences in development at one year of age between neonates with GS and control infants. (Table 4) Variables such as gestational age, duration of ventilation, commencement of feeds, establishment of full feeds and duration of stay in hospital were not associated with developmental delay. However, SGA neonates were found to have statistically significant delay in receptive and expressive language. (P less than 0.05) (Table 5)

**Figure:**
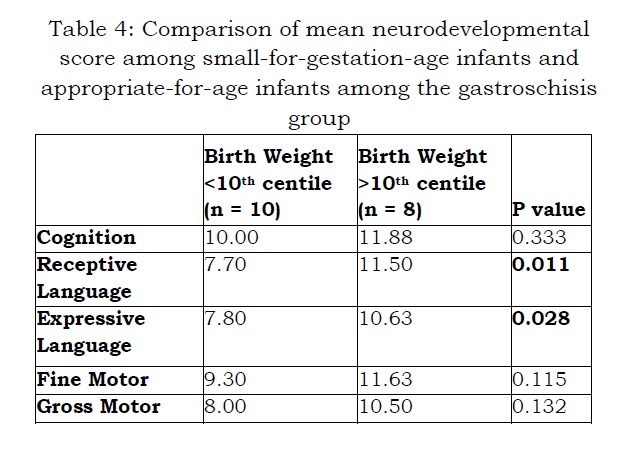
Table 4: Comparison of mean neurodevelopmental score among small-for-gestation-age infants and appropriate-for-age infants among the gastroschisis group

**Figure:**
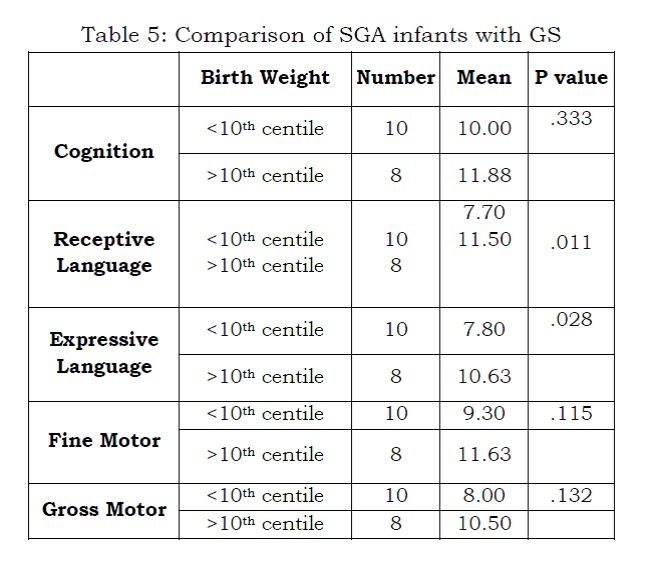
Table 5: Comparison of SGA infants with GS

## DISCUSSION

The primary objective of this study was to describe the neurodevelopmental outcome of neonates with GS. We found no statistically significant difference in the neurodevelopmental outcomes between neonates with GS and our matched controls. This finding was similar to that of Gorra et al. [17] who compared the neurodevelopment outcome of 46 neonates with GS at two years of age, although their study used an earlier version of the Bayley developmental assessment and excluded neonates with complex GS.

To date, there have been few studies focusing on the neurodevelopmental outcome of neonates. South et al. [13] used the Bayley II at follow up and found that only one infant had a score below average on the Mental Developmental Index. However, Henrich et al.’s [18] suggested that the initial delay is amenable to correction with close follow up and early intervention. A more recent study by Minutillo et al [19] used the Griffiths Developmental Assessment and Ages and Stages questionnaire at a 12-month follow-up of neonates with GS. This study revealed that only one infant had a low General Quotient and concluded that the incidence of adverse neurodevelopmental outcomes at one year of age appears to be low. A study by van Manen et al [20] is the only study published to date, which used the BSID-III as a developmental assessment tool. At 20 months of age, none of their patients had cognitive delay or cerebral palsy, a finding that is comparable to our study.

In this study neonates born with a birth weight less than 10th centile (SGA) did show a delay in both receptive and expressive language at one year of age compared with non-SGA infants. These neonates had significantly longer duration of hospital stay, prolonged ventilation, TPN dependence and more frequent TPN cholestasis, when compared with neonates with simple GS. There have been several studies in the last 10 years which identified comparable results.[18,19,21,22] Arnold et al. [23] included 4344 patients with GS and showed that babies with complex GS had a prolonged hospital stay and several comorbidities. Three of the four neonates, who were SGA at birth, remained so at their one-year follow up. 

Seventy percent of neonates had a primary closure as compared to a silo procedure followed by subsequent closure, which is comparable to other studies. [24] Although a mortality of 4.4% has been reported in other studies, there were no deaths in this cohort.

## Conclusion

Early neurodevelopmental outcome at one year of age in children with gastroschisis was not significantly different from that of control infants. However, small-for-gestational-age neonates with gastroschisis showed delay on both receptive and expressive language. Long-term follow-up is required for these infants as some delay may become apparent as they grow. 

## Footnotes

**Source of Support:** None

**Conflict of Interest:** None

